# Measuring what matters: Evaluating quality of life instruments through the eyes of people with Parkinson's disease

**DOI:** 10.1177/1877718X261480905

**Published:** 2026-10-08

**Authors:** Samantha Dorrance, Richa Patel, Glenn T Stebbins, Jamie L Adams, Allison Allen, Tara Hastings, Soania Mathur, Onanong Phokaewvarangkul, Gary Rafaloff, Rajasumi Rajalingam, Anette Schrag, Christine Sun, Jennifer Mammen, Connie Marras

**Affiliations:** 1 Edmond J. Safra Program in Parkinson's Disease and Morton and Gloria Shulman Movement Disorders Clinic, 568293 University Health Network, Toronto, ON, Canada; 2 Department of Neurological Sciences, 466502 Rush University Medical Center, Chicago, IL, USA; 3 Department of Neurology, 189452 University of Rochester, Rochester, NY, USA; 4 609772 Duke University Medical Center, Durham, NC, USA; 5 2194 Sanofi, Morristown, NJ, USA; 6 Parkinson's Research Advocate, Toronto, ON, Canada; 7 UnshakeableMD, Toronto, ON, Canada; 8 Chulalongkorn Centre of Excellence for Parkinson's Disease & Related Disorders, Department of Medicine, Faculty of Medicine, 26683 Chulalongkorn University and King Chulalongkorn Memorial Hospital, Thai Red Cross Society, Bangkok, Thailand; 9 13143 Parkinson's Research Advocate, Westlake, FL, USA; 10 Department of Psychiatry, 4175 Western Michigan University Homer Stryker M.D. School of Medicine, Kalamazoo, MI, USA; 11 Department of Clinical and Movement Neurosciences, 407078 UCL Institute of Neurology, University College London, London, UK; 12 14709 University of Massachusetts, Dartmouth, MA, USA; 13 7938 University of Toronto, Toronto, ON, Canada

**Keywords:** Parkinson's disease, patient-report, measurement, quality of life, qualitative

## Abstract

**Background:**

Many instruments are used to assess quality of life in people living with Parkinson's disease. However, there is limited understanding of what people living with Parkinson's consider relevant and important when measuring quality of life.

**Aim:**

To explore what people living with Parkinson's consider relevant and important in the assessment of quality of life.

**Methods:**

Semi-structured interviews were conducted with 31 people living with Parkinson's disease. Participants completed eight instruments, which were used to guide discussion and reflection on what helps and what hinders measuring quality of life in a way that is meaningful to them.

**Results:**

Participants highlighted a number of features they consider important in the assessment of quality of life. These include being able to select from a wide range of response options, addressing the personal, emotional, and practical impacts of issues, using clear, specific language, and including a balance of questions that address both positive and negative aspects of the Parkinson's experience.

**Conclusions:**

There is a need for instruments that reflect the complex and varied ways Parkinson's disease affects individuals. While existing questionnaires incorporate some features that support meaningful assessment, participants also identified features that may hinder the extent to which quality of life is adequately captured and that warrant further refinement. Incorporating feedback from people with lived experience may help inform future evaluation and refinement of quality of life measures in research and care.

## Plain language summary

Measuring What Matters: Evaluating Quality of Life Instruments Through the Eyes of People with Parkinson's Disease

Many questionnaires are used to assess quality of life in people living with Parkinson's Disease. However, there is limited understanding of what people living with Parkinson's disease consider important when measuring it. In this study, we aimed to better understand what features make a quality of life questionnaire meaningful and relevant to people living with Parkinson's. We conducted in-depth online interviews with 31 individuals living with Parkinson's. During the interview, participants completed eight questionnaires and shared their views on them. These questionnaires were used to guide discussion and help participants reflect on how quality of life should be measured. Participants provided feedback on what worked well and what could be improved when capturing aspects of quality of life that were important to them. They identified several areas for improvement, such as offering a wider range of answer options, addressing the personal, emotional, and practical impacts of issues, using clear, specific language, and including a balance of questions that address both positive and negative aspects of the Parkinson's experience. These findings help identify what people with Parkinson's value in quality of life measurement, and highlight the need for questionnaires that reflect the complex and varied ways Parkinson's affects individuals. While existing questionnaires have strengths, participants indicated that there is still room for improvement. Incorporating feedback from people with lived experience may help inform future evaluation and refinement of quality of life measures in research and care.

## Background

Quality of life (QoL) is a central outcome in Parkinson's disease (PD) research and care. In an era of increasing emphasis on patient-centered care and precision medicine, measures are expected to reflect outcomes that are meaningful to patients. This expectation is reinforced by regulatory guidance promoting the use of patient-informed measures.^1^

There is limited knowledge of what people living with PD (PwPD) consider relevant and important in the measurement of QoL. Within the field of PD, several instruments are used to assess QoL. Many instruments were developed based on interviews with PwPD^2–6^ and have undergone clinometric and psychometric validation.^2,4–8^ However, there is a lack of evidence directly derived from PwPD confirming that the resulting instruments capture QoL in a meaningful way. This gap highlights the need to better understand aspects of QoL assessment that are important from the PwPD perspective. To address this gap, we elicited feedback from PwPD on established instruments to identify patient-informed considerations for QoL assessment.

## Method

This study is grounded in the assumption that QoL is a subjective construct shaped by individual experiences, and that QoL in PD is best understood through the perspectives of those living with the condition. Accordingly, we conducted one-on-one semi-structured interviews with PwPD, during which participants reviewed and reflected on eight established QoL instruments. These instruments were used as prompts to elicit discussion about different aspects of QoL assessment. Participants were asked to comment on these instruments with the aim of understanding whether existing QoL assessments align with the patient perspective.

Interviews were conducted with flexibility to minimize participant burden. Participants could pause or stop at any time, take breaks, or split interviews across sessions. Interviewers monitored for signs of fatigue or distress and conducted brief check-ins following the review of each instrument. When distress occurred, the session was paused, and participants were supported to resume only if they wished; the principal investigator was notified and followed up with the participant as necessary.

This project was approved by the Research Ethics Board of the University Health Network.

### Instrument identification

Instruments used to evaluate QoL, wellbeing or life satisfaction in PD were identified from the following sources:

1. Clinical outcome assessment measures used in papers identified through an inclusive literature search of concepts associated with QoL in PD^9^
2. Instruments identified in a systematic review^10^
3. The 2011 MDS Critique and Recommendations of health-related quality of life instruments^11^
4. The Instrument Inventory held by the International Parkinson and Movement Disorders Society (https://www.movementdisorders.org/)
5. Instruments known to the authors. The authors comprise a group with expertise in movement disorders, clinimetrics and lived experience with PD. Hereafter they are referred to as the Expert Working Group.

A list of potential instruments was created and then distributed to the members of the Expert Working Group. This group was comprised of healthcare professionals, two PwPD and a former care partner. Using a consensus-based approach, working group members were asked to evaluate instruments for eligibility based on inclusion criteria (Table 1) and to identify any potentially missing instruments. Instruments that met eligibility criteria by consensus review were used for the next stage interview process.

**Table 1.** Instrument eligibility criteria.

Inclusion criteria	Exclusion criteria
English language	Intentionally assessing only symptoms
Patient-reported	Intentionally restricting to a particular problem or symptom domain OR intentionally excluding any domains.
Stated focus on QoL, life satisfaction or wellbeing	Generic measures
PD- or neurological disease-specific OR “Recommended or Suggested” in the 2011 Movement Disorders Society (MDS) Critique and Recommendations paper on Health-Related Quality of Life instruments for use in PD ^11^	

### Participant sampling and recruitment

PwPD were recruited from multiple sources between May 2023 and June 2024 to take part in the Phase 2 interviews. Advertisements were shared through Fox Trial Finder, the Michael J Fox Foundation online clinical study matching tool, and through Parkinson Canada support groups. Participants were also recruited through the Movement Disorders Clinic at Toronto Western Hospital. Eligibility criteria included clinical diagnosis of PD, age 18 years or older, ability to recall and report their PD symptoms, and English fluency. Immediately after enrollment, the MDS-UPDRS Parts I and II were administered, and participants were classified as having ‘mild’, ‘moderate’, or ‘severe’ disease using a previously published method^12^ (see footnote to Table 2). Partway through recruitment, to increase representation from moderate and severe groups, PwPD under the care of CM and from a list of former research participants at Toronto Western Hospital whose disease was more advanced were selectively invited to participate. Recruitment was concurrent with data collection and preliminary analyses in Phase 1; enrollment concluded when thematic saturation and rating stability had both been reached (see^9^). All participants provided written informed consent for study procedures.

**Table 2.** Participant characteristics (N = 31).

Ethnicity, N(%)	White	26 (84)
	Asian	4 (13)
	Black	1 (3)
Gender, N(%)	Male	18 (58)
	Female	13 (42)
Education, N(%)	Highschool	2 (6)
	College/University	29 (94)
Disease severity, N(%)	Mild^a^	26 (84)
	Moderate^b^- Severe^c^	5 (16)
Years since diagnosis, N(%)	3 or less	8 (26)
	4 or 5	6 (19)
	6 to 10	13 (42)
	more than 10	4 (13)
Age (median, IQR)		69, 10

Disease severity based on MDS-UPDRS scores:

a 1. Part II = 14–26 and Part I ≤ 26; or

2. Part II = 3–13 and Part I = 14–26; or

3. Part II = 0–2 and Part I = 14–26 and Item 1.1 ≥ 2

b 1. Part II = 27–39 and Part I ≤ 39; or

2. Part I = 27–39 and Part II ≤ 39

c 1. Part II > 39; and/or

2. Part I > 39

### Instrument review

One-on-one semi-structured interviews, each lasting between two and four hours, were conducted via Microsoft Teams over one to three sessions, depending on participant preference. Interviews were conducted with flexibility to minimize participant burden. Participants could pause or stop at any time, take breaks, or split interviews across sessions. Interviewers monitored for signs of fatigue or distress and conducted brief check-ins following the review of each instrument. When fatigue occurred, the session was paused, and participants were supported to resume only if they wished; the principal investigator was notified and followed up with the participant as necessary.

During these interviews, PwPD completed QoL instruments and provided feedback on each scale's strengths and limitations. Participants were asked to comment on the scale as a measure of QoL.

Each participant was first shown the list of concepts that they had previously (Phase I^9^) indicated as being “very important” to their QoL and that currently affect their QoL “a lot”. Then, participants completed each instrument that met our inclusion criteria. Immediately after completing each instrument, participants were asked “Consider what you have told us is important to your QoL. Taken together, do the items from the [instrument name] completely capture the impact of PD on your QoL?”. They responded using a rating scale from 1 (not at all) to 10 (completely). Participants were then asked an open-ended question about their overall impression of the scale, followed by a prompted discussion about that specific instrument's strengths and weaknesses in terms of concept coverage, item wording, answer choices, and any other features of the instrument that the participant would like to comment on. The complete interview guide can be found in the Supplemental Material.

### Data analysis

Interviews were audio recorded and transcribed using Microsoft Teams. Transcripts were exported into and analyzed using NVivo 14.0. Initial content coding was conducted by three study team members (RP, CS, SD), each responsible for coding the data of different participants, with NVivo used to support data management and organization. Responses to the question “considering that we are measuring QoL, does the item wording make sense to you?” were categorized as “makes sense,” “didn’t make sense,” or “ambivalent”. Similarly, responses to the question “considering that we are measuring QoL, do the answer options work for you?” were categorized as “works for them,” “doesn’t work for them,” or “ambivalent”, and responses to the question “did you experience any discomfort when answering these [the instrument's] questions?” were categorized as “yes” or “no”.

All participant feedback was analyzed using inductive thematic analysis. Each coder (RP, SD, CS) independently developed an initial codebook based on sample quotes and emerging themes. These preliminary codebooks were then reviewed, merged, and refined collaboratively with senior investigators (CM and JM). Through discussion, codes were merged, modified, expanded, and revised to reach consensus. The resulting codebook was then used as a foundation for subsequent coding. New codes were incorporated as they emerged, using a similar approach. A final review and refinement of the codebook and corresponding quotes were completed following the coding of the final participant's data.

## Results

### Participants

All participants (N = 44; 32 with mild PD and 12 with moderate-severe PD) from Phase 1 were invited to proceed to Phase 2. Thirty-one participated. One participant was deemed ineligible due to a revised diagnosis of progressive supranuclear palsy, three did not respond, four declined participation citing personal reasons (chronic conditions, anticipated stress related to the interview process or external stressors, lack of interest), and five were unable to participate in the interviews due to physical and emotional limitations. Participant demographics are presented in Table 2.

### Scales

Eight instruments for measuring QoL, life satisfaction or wellbeing in PD or neurological disorders were included (details in Table 3):

1. New satisfaction with Life and Treatment Scale in Patients with PD (SLTS-7)^13^
2. PD-Health Index (PD-HI)^14^
3. Belastungsfragebogen Parkinson Kurzversion (BELA-P-k)^7^
4. Parkinson's Disease Questionnaire - 39 items (PDQ-39)^3^
5. Parkinson's Disease Quality of Life Scale (PDQUALIF)^4^
6. Parkinson's Impact Scale (PIMS)^8^
7. Parkinson's Disease Quality of Life Questionnaire (PDQL)^5^
8. Quality of Life in Neurological Disorders (Neuro-QoL)^6^

**Table 3.** Overview of included instruments.

Scale	Stated purpose^a^	Scale development	# Items	Domains / structure	Response format
SLTS-7	Assesses satisfaction with life and treatment in PD.	Modified version of the SLS-6, to which 2 treatment questions were added, and from which the financial question was removed. The latter was removed because the financial situation has been shown to have a weak association with life satisfaction in previous PD research^13^ The SLS-6 was adapted from a scale used in student populations for the self-evaluation of overall satisfaction with life, and in 5 life areas: physical health, psychological well-being, social relations, leisure, and financial situation.^15^	7	Global life satisfaction; physical health; psychological wellbeing; social relations; leisure; treatment satisfaction; treatment expectations	1–10 rating scale (1 = not at all satisfied; 10 = very satisfied)
PD-HI^b^	Assesses symptoms and health-related quality-of-life from the perspective of PwPD.	“Developed using extensive patient input, including an analysis of 2978 patient quotes and a large cross-sectional study involving 404 patients.”^14^	96	Thirteen subscales: fatigue; sleep; mobility; activity participation; central sensory function; pain; gastrointestinal function; hand/arm function; social health; emotional health; cognitive function; communication; abnormal movements	5-point rating scale 0 = I don’t experience this to 5 = It affects my life severely
BELA-P-k	Measures psychosocial problems and need for help in PwPD.	Items derived from clinical observation; BELA-P-k created by item reduction. The Dutch version was reviewed by 10 patients for clarity and comprehension.^7^	19	Four subscales: achievement capability/physical symptoms, fear/emotional functioning, social functioning, and partner-bonding/family	Two 5-point rating scales per item: “Bothered by” (0 = not at all to 4 = a great deal), and “Need for help” [in the form of treatment] (0 = not important to 4 = very important)
PDQ-39	Assesses Health status in PwPD.	“Questionnaire items, generated from in-depth interviews with people with Parkinson's disease, were developed into a 65-item questionnaire.”^16^ Item reduction and validation were conducted using two postal surveys.	39	Eight scales: Mobility, Activities of daily living, Emotional well-being, Stigma, Social support, Cognition, Communication, and Bodily discomfort	5-point rating scale (0 = Never (i.e., no problem at all) to 4 = Always or cannot do at all (i.e., maximum level of problem))
PDQUALIF	Assesses disease-specific health-related quality of life in PwPD.	“Results from an initial qualitative study provided content for item development and scale construction.”^4^ This included responses from “52 PD patients, 28 spouses or significant others, and 6 PD professionals” to an open-ended question.	33	Seven domains: Social/role function, Self-image/sexuality, Sleep, Outlook, Physical function, Independence, and Urinary function. Plus one item of Global HRQoL.	5-point Likert-type response formats, including frequency, agreement, intensity, temporal frequency (past 7 days), and applicability-based scales.
PIMS	Measures the impact of PD on a patient's life.	“Designed by nurses in the Parkinson Foundation of Canada Clinical Assistance/Outreach Programmes.”^8^	10	Ten life areas (self; feelings; family; community; work; travel; leisure; safety; financial security; sexuality) across four factors: Psychological, Social, Physical, and Financial.	0–4 scale (0 = no change; 4 = most severe impact)
PDQL	Measures QoL in PwPD.	“An item pool was developed based on the experience of patients with Parkinson's disease and of neurologists; medical literature on the problems of patients with Parkinson's disease; and other quality of life questionnaires.”^5^ Items most often chosen and rated as important by patients were retained.	37	Four subscales: Parkinsonian symptoms, Systemic symptoms, Emotional functioning, Social functioning.	5-point rating scale (1 = All the time, 5 = Never)
Neuro-QoL	Measures health-related QoL (HRQoL) in adults and children with common neurological disorders (including Parkinson's).	“Relevant HRQL domains identified through a comprehensive review of the literature and literature search, expert interviews and surveys, and patient and caregiver focus groups.”^6^	101 (standard short forms) variable (CAT format; 13 items banks)	13 standard short forms across 4 domains: Physical Health: Upper extremity function-Fine Motor, ADL; Lower extremity function (Mobility); Fatigue; Sleep disturbance Emotional Health: depression; anxiety; stigma; positive affect and wellbeing; emotional and behavioral dyscontrol Cognitive health: applied cognition-general concerns; applied cognition – executive function Social health: ability to participate in social roles and activities; satisfaction with social roles and activities"	5-point Likert-type response formats, including frequency, difficulty, agreement, intensity, past-week frequency, and applicability-based response scales.

a The stated purpose of each instrument was derived from the original development and/or validation publications.

b The PD-HI has not yet been published.

The PDQ-8 was not included as a standalone instrument, as all items are contained within the PDQ-39.

### Participant insights on scale attributes

Participant feedback provided insights into four key aspects of QoL instruments: (1) framing of scale items, (2) scope of scale items, (3) answer choices, and (4) concept coverage. These aspects are explored in the sections that follow.

1. Framing of scale items
  With respect to the framing of scale items, participants commented on:
  - item valence (positive vs. negative);
  - Parkinson's disease–focused framing, particularly difficulty attributing issues specifically to PD; and
  - specification of the time period referenced.
  A major theme that emerged from narratives was that question framing matters (i.e., positive or negative valence). In general, positively-framed questions were viewed favorably, whereas negatively-framed ones were criticized for potentially causing distress. One potential participant declined participation anticipating emotional distress; another needed to pause the interview due to the distress induced by the instruments. This individual later recommended having a support person present for the respondent if they were not physically with staff, and to ask about emotional wellbeing after completing such instruments. PD-focused framing (“e.g., Due to Parkinson's disease … .”) was challenging due to the difficulty in attributing issues specifically to the condition. However, explicit instruction on whether to answer questions considering only PD or all aspects of life was preferred over leaving it up to interpretation. There were varying preferences regarding time period specification. Due to the fluctuating nature of PD both within and across days, determining ideal time frames for assessing QoL was challenging. Still, participants preferred that the time frame be specified rather than left to interpretation. They reported that longer time frames (one month and longer) posed challenges due to fluctuations in the condition, whereas shorter time frames (7 days and less) improved recall and allowed for assessment of their present condition.
  Exemplary quotes for these and related insights are presented in Table 4.
2. Scope of scale items
  With respect to the scope of scale items, participants commented on:
  - personal relevance;
  - the level of detail captured;
  - a preference for precise and specific wording; and
  - the importance of capturing both emotional and practical impacts of issues.
  Participants highlighted the individualized nature of QoL and expressed a desire for it to be captured accordingly. Some remarked that scale items should be customized based on what is important to the individual; several suggested achieving this by using an adaptive structure, in which high-level questions are followed by more specific ones, based upon previous responses. A preference was stated for detailed over general questions, which provide the opportunity to convey the complexity of one's personal experience with the disease. When items were relevant, lengthy scales were well tolerated due to their enhanced ability to capture the factors influencing the disease's impact on an individual's life. The value of asking about the practical and emotional impact of symptoms was also underscored.
  Exemplary quotes for these and related insights are presented in Table 5.
3. Answer Choices
  With respect to answer choices, participants commented on:
  - the effects of framing (positive vs. negative);
  - the importance of clear, unambiguous response options;
  - the need for an adequate range of anchors; and
  - the ability of response options to capture the extent of an issue's impact.
  The importance of positive framing was also conveyed with respect to answer choices. Positively framed options (e.g., 1 (not at all satisfied) to 10 (very satisfied) in the SLTS-7) were seen as encouraging a more hopeful reflection on one's condition, and were well received by participants. Negatively framed options (e.g., 0 (no change) to 4 (severe) in the PIMS) were thought to limit the nuance of one's responses by limiting the ability to convey positive aspects of their circumstances.
  Participants valued anchors that were specific and unambiguous, reducing the burden of interpreting vague terms. A wide range of options was favored in order to capture the range of experience possible in PD. Similarly, having a variety of anchors was appreciated, enabling participants to express their situation with greater accuracy and precision. For example, options ranging from “I don’t experience this” and “I experience this, but it does not affect my life” to “It affects my life severely”.
  Finally, participants emphasized the importance of response options that convey extent of impact. This helped to capture the personal impact of experiences beyond impairment or frequency of occurrence, such as effects on daily life and their meaning to the individual.
  Representative quotes are presented in Table 6.
4. Spontaneous reflections on concept coverage

**Table 4.** Exemplary quotes related to item framing.

Theme		Illustrative quotes
Valence	Positive	P11: The wording was somewhat more positive. You know, “I can,” “I am able” … There were more positive statements, but also “how much difficulty” … so there are negatives [too], we know that, we acknowledge it [but] it wasn’t all focusing on the negative. So there was a balance in that respect. [Neuro-QoL]
	Negative	P5: The results are saying, OK, you’re just sliding down, sliding down, sliding down. [PDQL] P9: I would want to be sensitive to somebody answering these questions … some of these are so bleak, “I felt worthless”, “I felt I had no reason for living”, “I felt that my life was empty.” Those are kind of provocative, [and] some people are despairing and so I think we have to be cautious … about answering these sorts of questions because it is a scary, scary disease. [Neuro-QoL]
Difficulty in restricting attribution of issues to PD	P5: It was just the fact that you couldn’t really separate whether it wanted you to talk about PD versus everything in your life. [SLST-7] P9: The thing is I can’t … differentiate [why] I'm feeling anxious … If I didn’t have Parkinson's disease, I might be every bit as anxious about my grandson. But on the other hand, maybe I wouldn’t be so anxious … I just don’t know whether it's Parkinson's. [PDQ-39]	
Optimal Time Period Specification	Shorter: better recall	P1: It said “in the last seven days” … and I thought, that's cool. That experience [is] what you felt recently as opposed to “past three months” … I couldn’t tell you what I felt a month ago. [PD-QUALIF]
	Longer: lacks sensitivity to present condition	P15: When they say “in the last day” [referring to another scale] … that makes it very clear. But when you start extending the period to … seven days [or] a month … [then] you can’t be as specific about the condition because you may … not have [an experience] in the first week, and then the second week … you’re bedridden … and then the third week you’re back to normal again. [Neuro-QoL]
	Longer: covers PD variations	P11: The thing I did like [was] “in the last month” … because it's … been an awful month … Again … it's fluctuation [PDQ39]

**Table 5.** Exemplary quotes related to item scope.

Scope	Personally relevant questions	P5: It's not always [about] asking more questions. It's trying to narrow it down so that you can keep the surveys relatively short and then ask more focused questions based on what the person thinks is important. [PD-QUALIF] P30: This [scale] was the first one to mention drooling [their most bothersome experience], so I gave it a high mark for that … it [the scale] “got” me. [PDQL] P24: They’re asking questions that don’t really give a good indication of QoL … like “difficulty swallowing” … I don’t think [questions like those] are a great measure of … what my QoL is. [PD-QUALIF] P23: You hit on just about everything that I have had to deal with [for] Parkinson's. [PDHI]
	Detailed questions	P26: I think the specificity is important because it’ll give whoever is evaluating a clearer picture of the person and their progression with the disease [Neuro-QoL] P9: The researchers [should be] getting … context about the living conditions of the person completing [the questionnaire] because I think that my circumstances are affecting my answers both positively and negatively. [PDHI] P47: I want both [cause and consequence] because without cause and effect, it's hard to relate how it impacts your life. [Bela-P-k]
	Preference for precise, specific question wording	P5: [if they ask] “Are you able to walk?” [but] don’t mention for how [long], I would say “yes”, but then that might cover more than one mile or less than one mile … I think it's much better to ask direct questions. [PDQL] P30: It's helpful that they’re [the items] specific because then we know what we’re dealing with [PDQ39] P15: The other [scales] were very, very specific [and] right to the point. And it's like “yes or no”. [In this scale] there's a lot of … “maybe” […] “gray area”, so it may not represent [QoL] … 100% the way you expect it to … Quality of life may … not [appear to] be impacted as much as the other questionnaires showed. [Neuro-QoL]
	Emotional and practical Impact of Issues	P9: I like the fact that there were … at least 2 dimensions there. One that was the dimension of impact [Bela-P-k] P27: I thought some of the questions captured how I feel on a daily basis. [ Bela-P-k] P6: It didn’t really assess your outlook on everything. It was more just again your symptoms. [PD-QUALIF] P19: I like how they’re asking very practical … questions … . “Are you able to turn the key in a lock”, “brush your teeth” … those are good functions to probe. [ Neuro-QoL]

**Table 6.** Exemplary quotes related to answer choices.

Theme		Illustrative quotes
Effects of framing	Positive	P11: It's a more positive way to look at things too[“1 (not at all satisfied)” to “10 (very satisfied)”] [SLTS-7]
	Negative	P28: The ability of the individual to speak positively about their condition is inhibited by the choice of [answer options :“0 (no change) to “4 (severe)”] [PIMS]
Need for unambiguous options	P4: Because I don’t have to sit here and decide what “sometimes” is, or what “often” is. Somebody's done that for me. [Neuro-QOL] P22: “Somewhat agree”, “agree” … [it] is not obvious to me [what] the difference [is]. [PDQ39]	
Need for a range of anchors	P31: It goes from basically “I don’t experience this” … all the way up to “it affects my life severely” … [and] the middle choices are pretty good [too]. [PDHI] P41: I like the fact there it says “cannot do it all” [PDQ-39] P28: It doesn’t offer any nuance in terms of explaining what affects me and what doesn’t. It's on or off, black or white, [but] … there are many aspects of PD that are more subtle. [Bela-P-k]	
Importance of capturing extent of impact	P30: Especially having both options of, “I don’t experience this” or “I experience this, but it doesn’t affect my life”. I think that was an important [distinction]. [PDHI] P30: The one about [not] being able to do up your buttons or dressing yourself, he said “occasionally”, but he first wanted to say “never” cause he says “you know, I'm slow, but I do it”. [Bela-P-k]	

With respect to concept coverage, participants spontaneously identified several important concepts as missing from existing scales.

When participants were asked to comment on ‘what is missing’, or what they liked and disliked about an instrument after having completed it, several common themes emerged. The comprehensive coverage of symptoms was highlighted as a positive feature. The inclusion of positive concepts (enhancers of QoL) was also viewed favorably. Notably, all of the concepts reported as missing by at least 5 participants were enhancers of QoL. These concepts related to emotions, attitudes and beliefs, as well as to social relationships and interactions (Table 7).

**Table 7.** Concepts reported as missing or insufficiently covered (by any scale) by at least 5 participants during the interview.

Concepts	Participants reporting as missing (N(%))
Support	15 (48)
Coping	11 (35)
Relationships	10 (32)
Involvement in decision-making	8 (26)
Knowledge of PD	8 (26)
Treatment	8 (26)
Gratitude	8 (26)
Access to healthcare	7 (23)
Empowerment	5 (16)
Faith	5 (16)
Financial concerns	5 (16)
Optimism	5 (16)

When asked about adding new concepts to a scale, participants explained that they did not expect every missing concept to be included. However, inclusion of positive factors in QoL assessments was viewed as critical to capturing important aspects of QoL. Several participants commented that omitting positive aspects like “coping” could lead to misinterpretation of responses, such as when scale items focus on “impairments” or “disabilities” rather than on their impacts. Illustrative participant quotes are presented in Table 8.

**Table 8.** Exemplary quotes related to concept coverage.

Theme	Illustrative quote
Positive factors as an inherent aspect of QoL	P21: I guess when I think of quality of life, I think of the balance between the negative and the positive. Like what do I […] bring to this to make my situation better or improve or manage my situation? Because I think that you can have a lot of negative things going on, but you can still have a positive attitude. P15: A lot of these [scales] focus on negative a little bit more than the positive … [but] you don’t flush out the fact that gratitude is quite … important as well for your quality of life. It may not help you get better, but it does help … the way you feel. [PD-QUALIF]
Excluding positive concepts increases risk of misinterpretation	P41: I started to say to myself “well, how often do I write?” I use my keyboard more often or I use my phone more often and I'll use … [speech-to-text tools]. So, we find coping [mechanisms] or shortcuts … to avoid those particular things, so we tend to minimize [the problems]. [PDQ39]

### Summary of scale features important to PwPD

Synthesis of key findings on what matters to PwPD with regards to QoL measures is presented in Table 9, with examples of effective vs. less effective implementation approaches.

**Table 9.** Synthesis of most important attributes for QoL measures with examples of optimal vs. suboptimal implementation.

Desired scale attribute	Effective implementation	Less effective implementation
Offers response options with a wide range of anchors	Providing response options that range from “I don’t experience this” and “I experience this but it does not affect my life” to “It affects my life severely” (PDHI)	Asking respondent to answer whether they experience something simply with “yes” or “no” (Bela-P-k)
Reflects personal level of impact	Providing response options that assess both presence of an issue, and the impact of this issue (PDHI) (see above)	Asks respondent about frequency of issues, e.g., “Never”, “Occasionally”, “Sometimes”, “Often”, “Always” or “Cannot do at all” (PDQ-39)
Reflects emotional and practical impact of issues	Asking about feelings, with items like “how positive you feel about yourself (self-worth, happiness, optimism)” (PIMS) Asking about function, with items like “Are you able to brush your teeth” (Neuro-QoL)	Asking about symptoms, with items like “I have aching/burning/coldness/numbness in my hand/feet” (PD-Qualif)
Uses clear and specific language, not requiring interpretation	Defining an experience precisely, with *scale items* like “During the past month due to Parkinson's disease, how often have you had problems *walking half a mile*?” (PDQ-39) Providing well-defined *response options* like: [In the past 7 days] … “never”, “rarely (once)”, “sometimes (2–3 times)”, “often (once a day)”, “very often (several times a day)” (Neuro-QoL)	Describing an experience broadly, with *scale items* like “… did you have trouble with *difficulties walking*” (PDQL) Providing ill-defined frequency options like: “never”, “rarely”, “sometimes”, “often”, “very often” (no descriptors) (Neuro-QoL)
Incorporates positive framing	Balance between *scale items* expressed in terms of retained function or ability, such as “I am able to do all of my regular family activities” and “I can keep up with my family responsibilities” and those expressed in terms of functional impairment or disability, such as “I felt unhappy” and “how much difficulty do you currently have learning new tasks or instructions” (Neuro-QoL) Similarly in *response options*, such as: [How satisfied are you with …] from 1 (not at all satisfied) to 10 (very satisfied) (SLTS-7)	All *scale items* framed negatively, or in terms “how often did you have trouble with …” (PDQL) All *response options* having a negative valence, such as “… what impact Parkinson's has had on your life, “0 = no change; 1 = slight; 2 = moderate; 3 = moderately severe; 4 = severe.”

## Discussion

In this study, PwPD reviewed a sample of instruments and provided feedback on approaches to QoL assessment. The findings contribute to an understanding of PwPD perspectives on QoL measurement in a way that reflects the QoL of an individual living with PD. The results highlight key features of effective approaches to assessing QoL as well as opportunities for improvement.

PwPD emphasize that instruments used to assess QoL should reflect personal levels of impact, include concepts that enhance QoL, offer response options with a broad range of anchors, and employ positive framing.

### Individualistic nature of QoL

The World Health Organization defines QoL as “an individual's perception of their position in life in the context of the culture and value systems in which they live and in relation to their goals, expectations, standards and concerns”.^17^ From this definition, it follows that the meaning one assigns to an experience is key to its relationship with their QoL. In our study, participants expressed appreciation for instruments that captured the personal impact of experiences. They responded favorably to wording that enabled them to convey this personal impact (e.g., “How much does the following impact your life now?” “I don’t experience this / I experience this, but it does not affect my life / It affects my life moderately / It affects my life very much / It affects my life severely”). These formulations acknowledge that the same experience can impact people differently,^9,18,19^ or shift over time within an individual^20^ depending on factors such as attitude, coping strategies, personal preferences, or lifestyle choices. These findings also suggest that individual differences in psychological characteristics, such as coping style, resilience, or psychological flexibility, may contribute to how people interpret and respond to QoL questions, even when clinical disease severity is similar.

Recognizing this individual variability highlights the need for more personalized approaches to QoL assessment. One approach is to allow individuals to nominate issues of importance to them. The Schedule for the Evaluation of Individual Quality of Life - Direct Weighting (SEIQoL-DW)^21^ allows individuals to list and rank their top five life areas contributing to wellbeing, and rate their satisfaction with each. Another approach is computerized adaptive testing (CAT), in which questions are presented based on a respondent's prior answers to tailor the assessment to their individual priorities. These models have demonstrated success in various healthcare domains, including cardiac rehabilitation,^22^ orthopedic care,^23^ and neurology,^24^ and have been validated for use in assessing generic health-realted QoL.^25^ To our knowledge, no CAT models have been applied to measuring QoL in PD, and the Neuro-QoL, although capable of being administered as a CAT, has not been tested as a CAT in PD populations.^26^ CAT may be particularly useful in PD given its multi-system and heterogeneous nature, which requires broad item coverage despite many issues being relevant to only a subset of individuals. By adapting questions to the individual, CAT offers a practical means of capturing the subjective and individualized nature of QoL highlighted by our findings.

### Importance of positive factors and framing

Participants’ reporting experience was negatively affected by the general lack of positive factors and framing in the instruments. They expressed concern about the predominantly negative framing of items and response options, which is a critique that echoes concerns raised in previous QoL work.^27^ Negatively framed questions appeared to heighten participants’ awareness of their illness and portrayed their condition in an overly distressing manner. This finding is important because it suggests that the way items are framed can directly influence how participants experience and respond to instruments, highlighting a need for clinical outcome assessments to incorporate positive factors and balanced framing. Including positive concepts, using more neutral wording or avoiding unnecessarily negative wording, and incorporating prompts that allow participants to indicate distress or a need for emotional support may help mitigate these negative effects. Striking a balance between positive and negative perspectives could enhance participant engagement and retention in research studies.

It has also been previously noted that instruments used to measure QoL tend to overemphasize clinical and functional outcomes while overlooking subjective, patient-centered dimensions of wellbeing.^28^ This perspective was echoed in participants’ comments, which emphasized that positive experiences are fundamental to QoL and should be incorporated into its assessment. Elements such as social support, coping mechanisms, and gratitude were frequently identified by participants as missing yet meaningful contributors to their overall QoL during the interviews. This is consistent with other work; among 123 PwPD interviewed using the SEIQoL-DW, it was found that family, health, marriage, and friendships were rated as essential to QoL by at least 30% of their participants.^21^ More broadly, research suggests that positive experiences can help individuals maintain a high QoL, even as physical health declines.^29^ Thus, in addition to enabling a more accurate assessment of an individual's QoL, identifying and evaluating QoL enhancers can yield insights that would support a more holistic, patient-centered approach to care.

### Quality of life vs health status

The distinction between QoL, health-related QoL and health status remains an area of ongoing discussion.^18^ Health-related QoL is generally understood as the dimension of QoL that focuses on the impact of health status.^30^ While health status refers to the clinical features of an individual's illness as reported by the individual, health-related QoL refers to how they perceive and react to it. Being closely related, these constructs have been conflated in the literature, with a review finding that most studies claiming to measure health-related QoL in PD primarily assess health status instead.^31^ In our study, though participants appreciated instruments that covered a broad range of personally relevant PD symptoms, underscoring the importance of health status, they emphasized the added value of being asked about its emotional and practical impacts, pointing to a conceptual difference between health-related QoL and health status. This observation supports the view that while evaluations of health status may approximate health-related QoL, truly capturing it requires instruments that consider the individual's subjective, internal experience rather than only the presence of symptoms and their impact on function.

### Our results in context

The last time QoL assessment for PD was evaluated and considered for possible refinement was in 2011, when a task force convened by the Movement Disorder Society (MDS) conducted a systematic review of instruments used to assess health-related QoL in PD.^11^ The Task Force emphasized that each instrument had limitations and advised that scale features be carefully considered in the process of selecting an instrument for a given context of use, but ultimately determined that the development of a new instrument was not warranted.^11^ Our study complements the findings of the 2011 MDS Task Force by providing a new lens through which to consider and evaluate QoL instruments: that of the person with lived experience.

One of the core challenges in measuring QoL is the absence of a universally accepted definition. Given that QoL is inherently subjective, eliciting feedback from PwPD offers a way to assess whether existing instruments reflect the construct as PwPD themselves understand it. Additionally, the qualitative nature of our study further allowed us to explore which features of an assessment make it meaningful and relevant from the PwPD point of view, offering useful insight into what works best in QoL measurement.

### Limitations

Several limitations should be noted. First, the order in which the instruments were presented to participants during their interviews was not randomized across participants, which may have introduced response bias. Attrition was another challenge, particularly during the second part of the study. Due to the lengthy nature of the interviews, some participants withdrew from the study before completion. Dropout was particularly common among participants in the moderate-severe group, resulting in limited representation of individuals with more advanced PD. Consequently, stage-specific analyses were not undertaken. A larger and more balanced sample across disease stages would improve understanding of how perspectives may vary.

The applicability of QoL tools across cultural contexts is an important consideration. Attributes of QoL instruments, as well as how QoL is conceptualized and assessed, may vary across populations and settings. Given the small sample size and the predominantly white, English-speaking Canadian participants, we were unable to examine how broader contextual, cultural, clinical, socioeconomic, and psychological factors may shape perceptions and experiences of QoL in PD. Consistent with the World Health Organization's framework, future instrument development and validation studies should seek to recruit participants across a broad range of sociodemographic, cultural, clinical, and socioeconomic circumstances. Collecting information such as migration history, language proficiency, ethnicity, healthcare access, social support networks, employment and financial circumstances, disease characteristics, psychiatric comorbidities, treatment experiences, and baseline psychological characteristics (e.g., coping style, resilience, optimism, baseline anxiety and depressive traits, and psychological flexibility) may help clarify how contextual, clinical, socioeconomic, and psychological factors influence the experience and assessment of QoL in PD. Future studies should also explore whether these factors moderate the relationship between disease experiences and QoL outcomes, which may help explain variability in QoL among individuals with similar clinical presentations and inform the interpretation, validation, and application of QoL measures across diverse patient populations.

### Moving forward

From the perspective of PwPD, current measurement tools have strengths, but also areas needing improvement. Though this study does not evaluate instrument performance or adequacy, its findings highlight aspects of QoL assessment that would be important when considering how QoL is measured in PD. These insights may provide a foundation for future efforts to re-examine or refine existing instruments. Key attributes to consider should be the ability to capture and convey personal levels of impact, inclusion of concepts that enhance QoL, including response options with a wide range of anchors, and the application of positive framing. Together, these findings contribute patient-centered considerations that should inform efforts to evaluate existing instruments and improve the assessment of QoL in PD.

## Supplemental Material

sj-docx-1-pkn-10.1177_1877718X261480905 - Supplemental material for Measuring what matters: Evaluating quality of life instruments through the eyes of people with Parkinson's disease Supplemental material, sj-docx-1-pkn-10.1177_1877718X261480905 for Measuring what matters: Evaluating quality of life instruments through the eyes of people with Parkinson's disease by Samantha Dorrance, Richa Patel, Glenn T Stebbins, Jamie L Adams, Allison Allen, Tara Hastings, Soania Mathur, Onanong Phokaewvarangkul, Gary Rafaloff, Rajasumi Rajalingam, Anette Schrag, Christine Sun, Jennifer Mammen and Connie Marras in Journal of Parkinson's Disease

## Data Availability

The coded data is available upon request from the authors. Original transcripts cannot be shared for privacy reasons.
