# Supplementary material for: Measuring what matters: Evaluating quality of life instruments through the eyes of people with Parkinson's disease
Source: J Parkinsons Dis. 2026 Oct 8:1877718X261480905. Online ahead of print. doi: 10.1177/1877718X261480905 (PMC13650055; doi:10.1177/1877718X261480905)
Supplement: Supplementary file 1 [file sj-docx-1-pkn-10.1177_1877718X261480905.docx]

Supplemental Material

[Interview Guide	22](#_Toc211590598)

# **Interview Guide**

1. **What was your overall impression of the questionnaire?** You rated the questionnaire xx out of 10 – Why?
   a. What did you like? (good attributes)
   b. What did you not like? (identify specific issues)
   c. How could it be changed to better capture the impact on your QoL?
2. **Considering that we are measuring QoL, does the item wording make sense?**
3. **Considering that we are measuring QoL, do the answer options work for you?**
4. **Did you experience any discomfort when answering these questions?**
5. **These are the concepts that you indicated were important to your quality of life and that are currently affecting you. Are there any that were not adequately covered?**
   a. If concept is not present – Do you think this is a problem?
